# Diagnostic challenges and therapeutic approach for aortic pseudoaneurysm infection with *Paenibacillus pueri*: a case report

**DOI:** 10.1128/asmcr.00229-25

**Published:** 2026-04-30

**Authors:** Jacob Owens, Lauren Jenkins, John S. Stevenson, Cindy Schmidt, Jasmine R. Marcelin

**Affiliations:** 1 Internal Medicine Residency, College of Medicine, University of Nebraska Medical Center 12284, Omaha, Nebraska, USA; 2 Plastic Surgery Residency, The University of Texas Health Science Center at Houston (UTHealth Houston) 12340, Houston, Texas, USA; 3 Department of Pathology, Microbiology, and Immunology, University of Nebraska Medical Center 12284, Omaha, Nebraska, USA; 4 Leon S. McGoogan Health Sciences Library, University of Nebraska Medical Center 12284, Omaha, Nebraska, USA; 5 Department of Internal Medicine, Division of Infectious Diseases, University of Nebraska Medical Center 12284, Omaha, Nebraska, USA; Vanderbilt University Medical Center, Nashville, Tennessee, USA

**Keywords:** *Paenibacillus*, *Bacillus*, aortic pseudoaneurysm, MALDI-TOF

## Abstract

**Background:**

*Paenibacillus* species are rod-shaped, gram-variable, endospore-forming, environmental bacteria. Once classified within *Bacillus* spp., it was reclassified based on 16S rRNA sequencing. *Paenibacillus* spp. has rarely been found to be pathogenic in humans; however, recent reports have been described in immunocompromised patients.

**Case Summary:**

A 60-year-old male with past medical history of hypertension, hyperlipidemia, atrial fibrillation, and bicuspid aortic valve presented to an outside hospital for 3 days of chest pain without associated symptoms. He was transferred to our institution after computed tomography (CT) demonstrated fluid around the aortic root concerning for aortic pseudoaneurysm. Admission and follow-up blood cultures were negative. He was started on ceftriaxone and vancomycin and underwent aortic root repair. Intraoperative cultures grew gram-indeterminate bacilli identified as *Paenibacillus pueri* by 16S broad-range PCR at an outside institution. Initial pathogen identification was complicated without discernible morphological differences between *Paenibacillus* spp. and *Bacillus* spp. Final identification often relies on molecular methods. Previous cases utilized amoxicillin-clavulanate or trimethoprim-sulfamethoxazole, with resistance demonstrated to penicillins and clindamycin. In our case, doxycycline was chosen based on limited susceptibility profiles and considering antibiotic side effect profiles.

**Conclusion:**

This is the third documented human case of *Paenibacillus pueri* infection. *Paenibacillus* spp. infections have varied clinical presentations, typically described in immunocompromised patients. This case demonstrates the difficulties in identifying *Paenibacillus pueri*. While rare, *Paenibacillus* spp. has been occasionally implicated in human disease. Even in immunocompetent patients, suspicion for true pathogens should remain high with repeated isolation on separate specimens.

## INTRODUCTION

*Paenibacillus* is a group of rod-shaped, gram-variable, endospore-forming bacteria (1, 2). These are often environmental bacteria with roles in promoting plant root growth, nitrogen fixation, bioremediation, and phosphate solubilization, leading to their use in biofertilizers (1, 3). Additionally, they play a role in producing antimicrobials and inducing resistance in plant tissues that neutralize phytopathogens (1, 4, 5). In particular, *Paenibacillus polymyxa* is a key species in producing the polymyxin antimicrobial class that has been key in fighting both human and plant pathogens (6–8). In humans, recent infections have been reported generally in immunocompromised individuals (9). We present a novel case of an infected aortic pseudoaneurysm within an immunocompetent patient.

## CASE PRESENTATION

**Fig 1:**
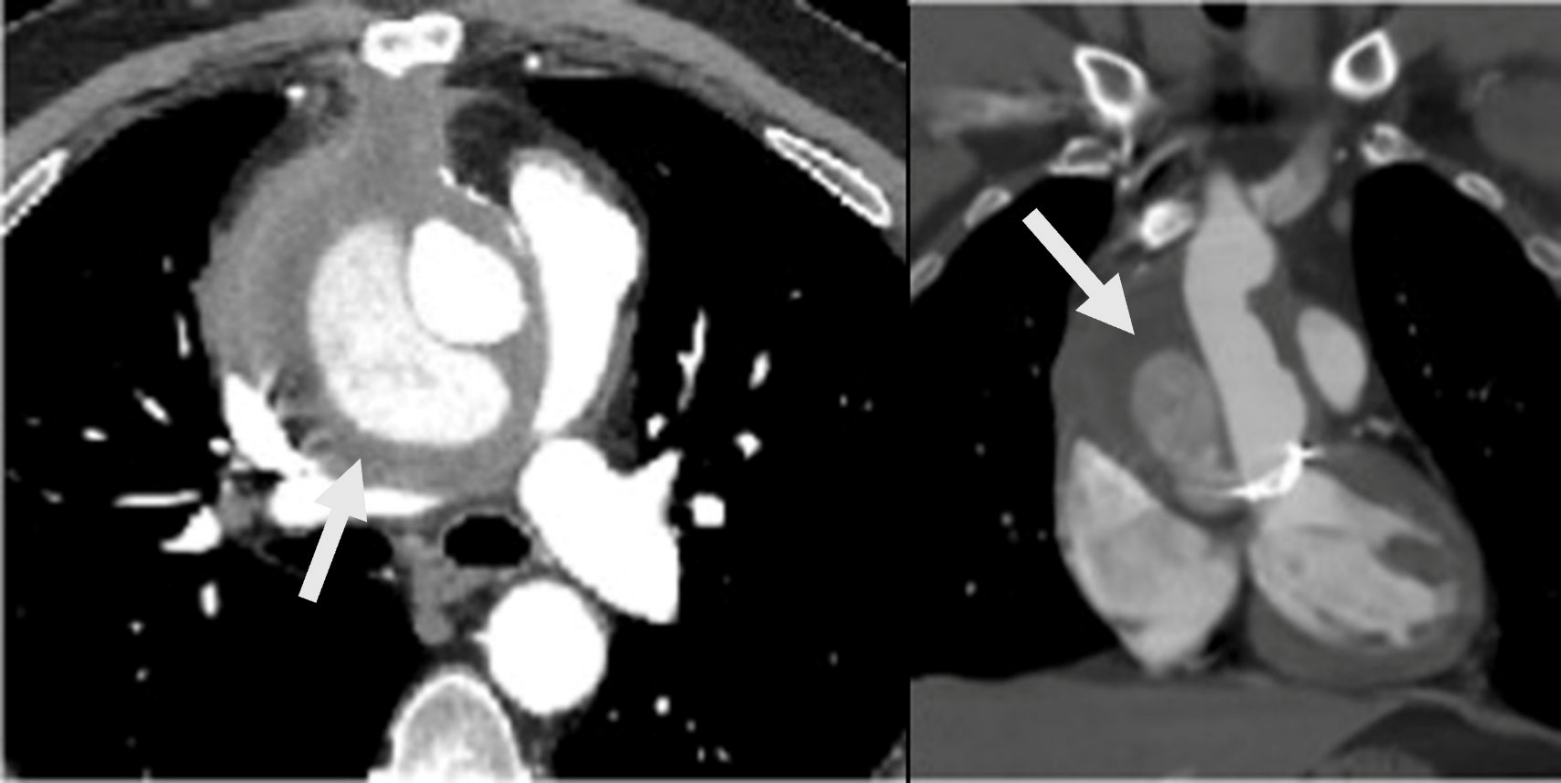
Pseudoaneurysm CTA. Initial chest tomography angiography axial (left) and coronal (right) views. There is a dilated ascending thoracic aorta and aortic root, measuring up to 7.6 × 7.2 cm transaxially, with aortic dissection extending from the aortic root to the mid-t-distal ascending thoracic aorta, with associated opacification of the false lumen with contrast. Additional intramural and extramural hematomas are visualized, concerning for pseudoaneurysm vs rupture vs impending rupture, requiring further evaluation by cardiothoracic surgery (arrows).

A 60-year-old male with a medical history of hypertension, hyperlipidemia, atrial fibrillation on anticoagulation, and myocardial infarction status-post-thrombectomy in 2014 presented to his local emergency department with 3 days of persistent substernal chest pain. He had additional history of prior mechanical aortic valve and root replacement in 2014 for bicuspid aortic valve. He reported no dyspnea, diaphoresis, or palpitations. He also denied fevers, chills, or weight loss over recent weeks. Social history included living on an acreage with his wife, performing his own landscaping, owning two cats, and having a 15-pack-year tobacco history. On examination, the patient was afebrile and tachycardic, with the only positive findings being an abnormal heart rhythm and mechanical valve sound. While electrocardiogram was negative, chest computed tomography angiography (CTA) demonstrated a dilated ascending thoracic aorta (7.2 × 7.6 cm) with ascending aortic dissection and intramural and extramural hematomas concerning for aortic rupture vs pseudoaneurysm (Fig. 1).

**Fig 2:**
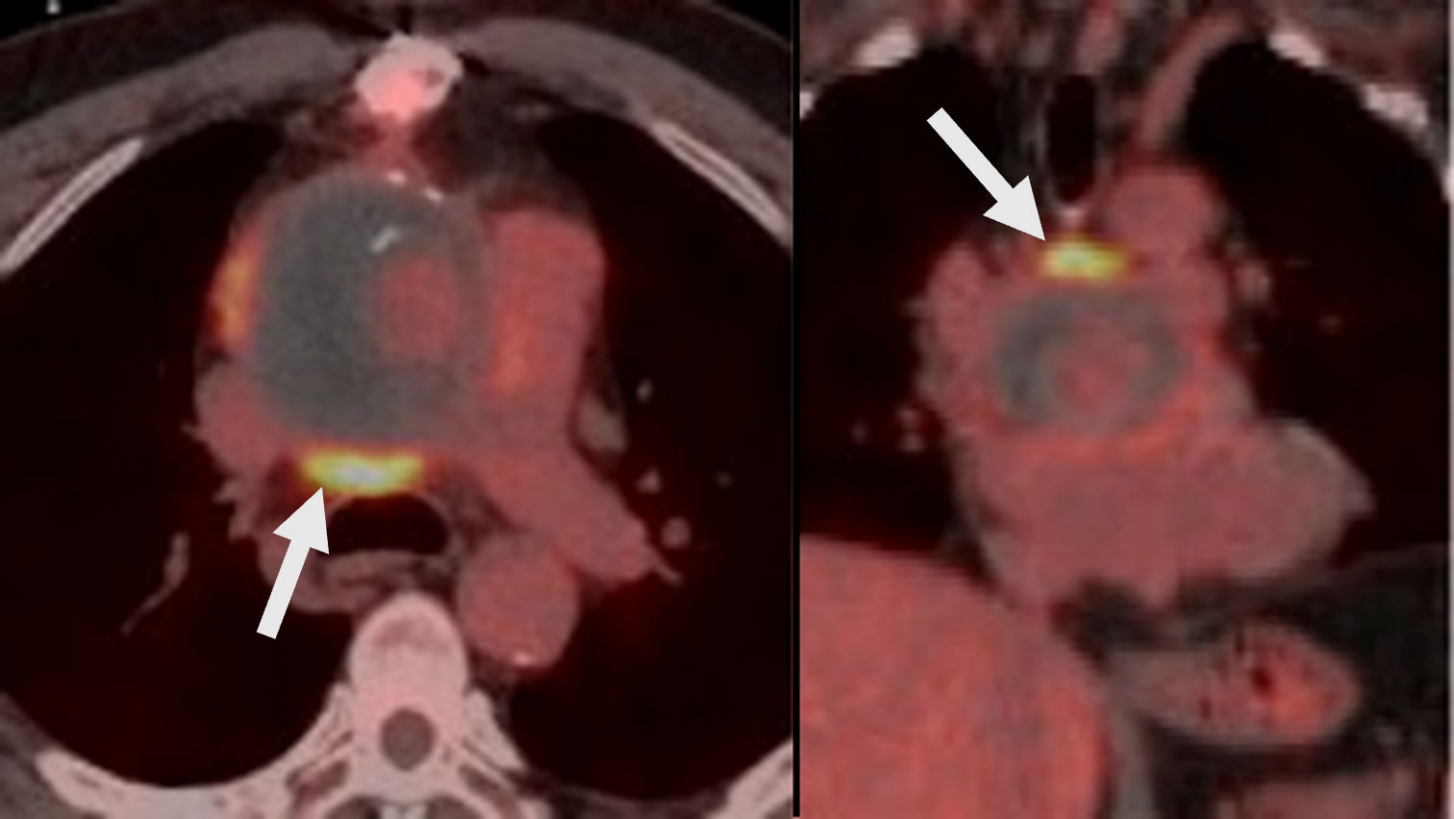
Pseudoaneurysm PET/CT. F-18 FDG PET/CT axial (left) and coronal (right) views. Depicts postoperative imaging following aortic root repair with fluid collection surrounding the aortic root. Along the periphery of this collection, there is avid FDG uptake (arrow) with SUV maximum up to 10.5, potentially inflammatory, though highly suspicious for infection given the degree of FDG uptake.

**Fig 3:**
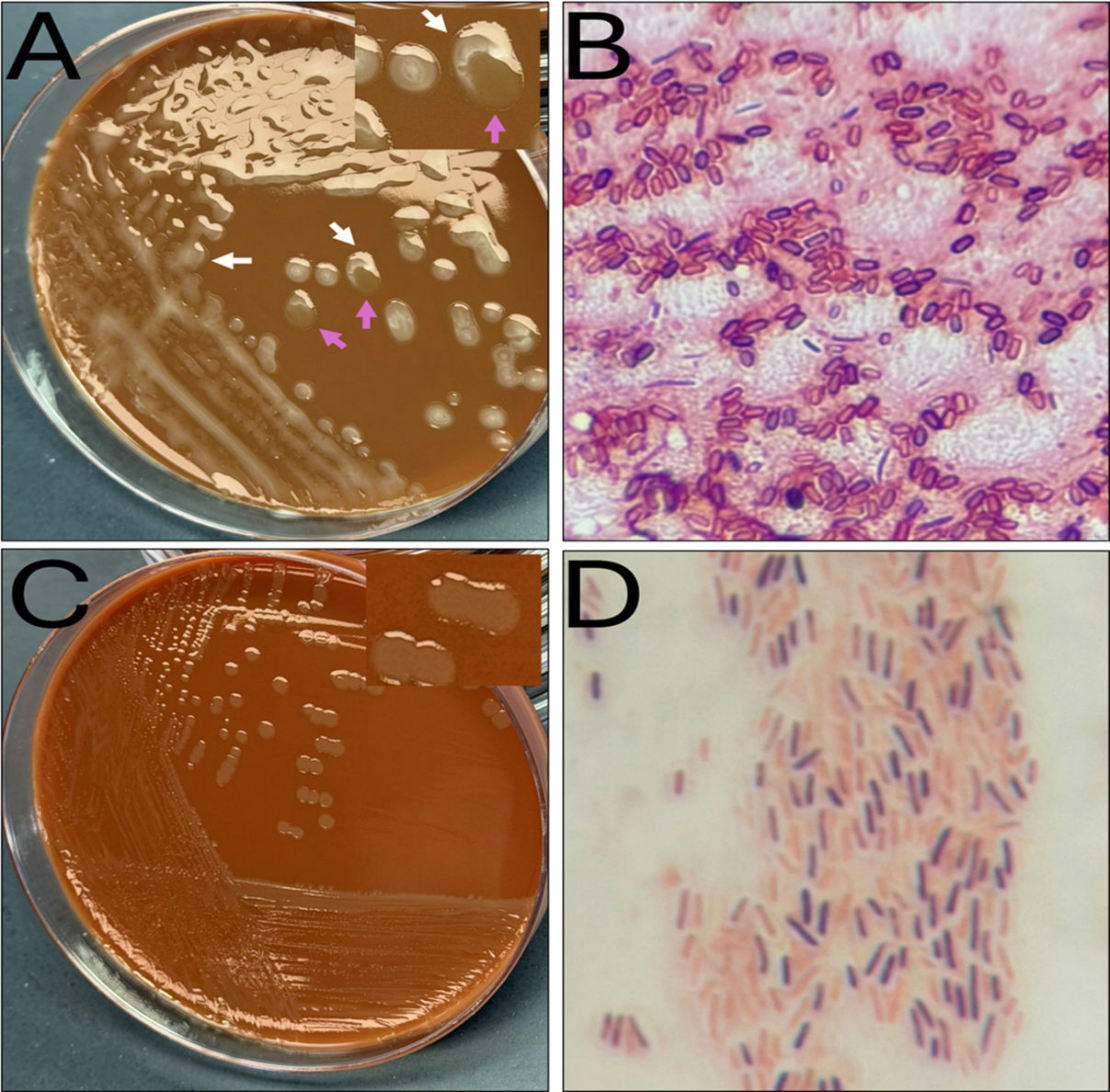
Colony morphology and Gram stain of the *Paenibacillus pueri* isolated from the mediastinal culture. (**A**) Representative colony morphology on chocolate agar showing a shiny white phenotype (marked by white arrows) that is morphing into a flatter colony morphology (pink arrows). The inset is a close-up view of the same agar plate. (**B**) The Gram stain from panel A showing both gram-negative and gram-positive central endospores and bacilli forms. (**C**) 72-h subculture from panel A with the inset showing flat colony morphology. (**D**) The corresponding Gram stain from panel C showing both gram-negative bacilli and gram-positive bacilli features. These studies were performed on a saved freezer isolate originating from the mediastinal culture.

Due to these imaging findings, the patient was transferred to our institution for evaluation by cardiothoracic surgery. F-18 fludeoxyglugose (FDG) positron emission tomography (PET)/CT demonstrated a fluid collection at the aortic root with increased FDG uptake concerning for infected pseudoaneurysm (Fig. 2). Following this result, the patient was initiated on empiric ceftriaxone and vancomycin and underwent re-do sternotomy and aortic root replacement with placement of a Dacron graft. Intraoperative fluid, aortic, and mediastinal tissue cultures were taken, and the initial Gram stain from all three surgical specimens showed “few-to-many white blood cells, many red blood cells, but no organisms seen.” The subsequent culture results from the intraoperative fluid (pseudoaneurysm hematoma), aortic tissue culture (pseudoaneurysm wall), and mediastinal tissue culture were all positive for a gram-variable rod resembling a *Bacillus* species. Occasional central endospores were also noted from the colony Gram stain. Figure 3 depicts the colony morphology and Gram stain characteristics of the bacteria isolated from the mediastinal tissue culture.

### Microbiology studies

To further identify the clinical isolates, we utilized the MALDI-TOF Biotyper Clinical Applications (CA) System (Bruker, Billerica, MA, USA; FDA-approved Reference Library Claim 6 spectral database and Research Use Only [RUO] MBT Compass Library Revision K [2022] spectral database). The MALDI-TOF was unable to identify the isolates using the FDA-approved CA Claim 6 spectral database. However, identification as *Paenibacillus pueri* (DSM 22970^T^) was obtained using the RUO, non-FDA-approved spectral database. Taking into consideration the Gram stain and colony morphology and combining those features with the RUO MALDI-TOF results, it was decided to report the isolates as “Gram-variable rods, most closely resembling *Bacillus* species, not *anthracis*” in accordance with our standard operating procedure.

The observation that all three of the surgical specimens grew “Gram-variable rods most closely resembling *Bacillus* species, NOT *anthracis”* raised clinical suspicion for a true pathological process rather than contamination, supporting this organism as the likely etiologic agent. Therefore, aortic tissue (pseudoaneurysm wall) was sent for broad-range PCR targeting the 16S ribosomal RNA gene (rRNA) with reflex to next-generation sequencing (NGS) at the University of Washington Department of Laboratory Medicine and Pathology (Seattle, WA, USA, Lab Code BCTDNA). Concomitantly, the gram-variable rod, most closely resembling *Bacillus* species, not *anthracis* isolated from the mediastinal culture, was sent to the Associated Regional and University Pathologist, Inc (ARUP, Salt Lake City, UT, USA) for aerobic organism identification (partial 16S rRNA sanger-sequencing, Lab Code 0065070) with reflex to susceptibility testing. All blood cultures obtained (a total of four sets of paired aerobic and anaerobic bottles) were negative after 5 days of incubation on an automated blood culture system (BD BACTEC, Sparks, MD). Additional infectious work-ups including Q fever serology, Histoplasma serum and urine antigens, and *Bartonella* serology were all negative. During the rest of the admission, the patient remained afebrile and clinically stable and was ultimately discharged on IV ceftriaxone 2 g daily and vancomycin 1,500 mg twice daily 15 days after the initial presentation.

Several days post-discharge, the aerobic identification by partial rRNA gene sequencing report from ARUP resulted as “Gram Positive Rods, Unable to definitively identify organisms by partial ribosomal DNA sequencing using current quality-controlled databases. Sequence most closely related to: *Paenibacillus species*.” Interestingly, the University of Washington also had similar difficulties: “Multiple bacterial DNA templates detected, which cannot be resolved by standard Bacterial PCR. Evaluating sample for reflexive NGS testing. Results to follow, see separate report.” The reflexive report for NGS testing was read as “[Minor abundance]: *Paenibacillus pueri*, Bacteria of the Family *Paenibacillaceae* (see comment). Comment: Public databases are not adequate to identify this organism. Most likely a previously undescribed species.” The difference between the sequencing methods can be attributed to full-length rRNA sequencing by NGS vs partial rRNA gene sequencing (10, 11).

### Treatment and follow-up

Susceptibility data for different antimicrobials are included in Table 1. Given the location with a new graft placed in an infected space and growth of the organism in three separate cultures, prolonged suppression therapy with repeat imaging was recommended. After reviewing the minimum-inhibitory concentration (MIC) breakpoints and the adverse effect profiles of the different treatment options, the decision was made to switch to oral doxycycline 100 mg twice daily to mitigate the risks of prolonged IV antibiotic treatment. Four months after patient discharge, he underwent repeat imaging with cardiac CT and F-18 FDG PET/CT. This demonstrated a stable fluid collection with proximal aortic endograft repair with FDG uptake decreased relative to initial PET CT, reassuring for resolving infection. Following a shared decision-making discussion regarding treatment and concerns for increased photosensitivity, the decision was made to discontinue doxycycline therapy after 1 year and transition to routine clinical monitoring, with no evidence of recurrent infection 6 months later at final ID follow-up and 2 years later at the subsequent cardiology follow-up.

**TABLE 1:** *Paenibacillus pueri* susceptibility results^*a*^

Antibiotic	MIC (µg/mL)	Interpretation
Ceftriaxone	≥4	No interpretation
Clindamycin	2	Intermediate
Doxycycline	≤0.25	No interpretation
Erythromycin	1	Intermediate
Gentamicin	≤2	Susceptible
Levofloxacin	≤0.25	Susceptible
Linezolid	1	No interpretation
Meropenem	0.25	Susceptible
Penicillin	4	Resistant
Trimethoprim-sulfamethoxazole	0.12/2.4	Susceptible
Vancomycin	16	Non-susceptible

^*a*^ *Paenibacillus pueri* MIC data from reference lab using lyophilized broth microdilution panels with interpretative breakpoints based on CLSI M45 ED3, Table 4 (12). *Bacillus *spp. (not *Bacillus anthracis*) and related genera (related genera include *Brevibacillus, Cohnella, Lysinibacillus, Paenibacillus, *and* Sporolactobacillus*).

## DISCUSSION

To our knowledge, this is the third documented case of human infection by *Paenibacillus pueri*. The organism has been previously isolated in blood samples from cases in Spain and Taiwan (2, 13). Originally, the organism was isolated from Pu’er tea, which gave it its namesake (14). While previous literature has documented the isolation of this organism from blood samples, with one being specifically defined in a patient with bacteremia (13), this is the first case of a pseudoaneurysm infection with *Paenibacillus pueri*. Additionally, our patient was immunocompetent, while this genus has previously been reported in immunocompromised individuals (9). While the patient had no known risk factors for infection, his history of mechanical valve placement and aortic root replacement may have served as possible risk factors for infection. His history of landscaping and gardening may have potentially introduced this normally environmental bacterium; however, the location of infection is still peculiar, and no definite entry point has ever been determined in this case.

Preliminary laboratory research has been presented implicating this genus among other bacteria as possible pathogens contributing to biofilm formation and subsequent lung inflammation and modulation within cystic fibrosis patients (15, 16). Furthermore, as human pathogens, other species including *P. macerans, P. thiaminolyticus, P. alvei, P. urinalis, P. lautus, P. phoenicis, P. timonensis, P. provencensis,* and *P. glucanolyticus* have been documented in a wide variety of diseases, including bacteremia, skin and soft-tissue infections, infected pseudoaneurysm, endocarditis and septic emboli, meningitis, post-infectious hydrocephalus, lung abscesses, and bacterial peritonitis (2, 17–28). Our case presents the first documented case of a *Paenibacillus*-infected aortic pseudoaneurysm. Similar to previous cases, our work-up was complicated by difficulties with species-level identification by FDA-approved methods or LDTs, which required the isolates being sent to an outside laboratory.

*Paenibacillus* was once included within the *Bacillus* genus but was later reclassified due to phylogenetic differences (1, 14, 29, 30). Identification as a *Bacillus* species or related *Paenibacillus* genera is not difficult, but definitive identification to the species level can be a challenge due to its morphological similarity to *Bacillus species*. Previous cases have also shown that biochemical testing is unreliable for identification of *Paenibacillus* spp., leaving MALDI-TOF (using FDA-approved and/or RUO spectral databases) or LDTs with public databases as the main diagnostic test for species-level identification, with varying results (17, 21, 31). In our case, our institution’s microbiology lab was unable to definitively report the identification of the clinical isolates. Due to the high clinical suspicion for a pathological process, we sent the aortic tissue and the mediastinal isolate for further identification by partial 16S rRNA testing, modalities considered LDTs. Surprisingly, both outside laboratories were unable to identify these bacteria to the species level by partial (~500 bp) rRNA gene sequencing. It was only after full-length rRNA gene sequencing by NGS (~1,500 bp) that a species-level identification was made. This suggests that for uncommon clinical isolates, full-length rRNA gene sequencing might have the best success for species-level identification. Not all institutions have access to such LDTs, however, and a reported result with an unspecified *Bacillus* species could prompt concerns for contamination versus true infection. However, our case demonstrates that when there are multiple culture results demonstrating repeated isolation, clinicians should have a high suspicion of a true pathogen, even without species identification, and pursue further work-up so that appropriate targeted therapy may be initiated.

Previous *Paenibacillus* cases have reported success with ceftriaxone, amoxicillin-clavulanate, and trimethoprim-sulfamethoxazole (17, 18, 21). Following identification of *P. pueri* and reviewing the MIC data provided from the outside laboratory (Table 1), the decision was made to initiate doxycycline monotherapy as it had the lowest MIC and presented a rational oral regimen with a more favorable adverse effect profile for prolonged treatment. However, even with these MIC data, no susceptibility interpretation was available for some antibiotics because the clinical breakpoints do not exist (12). Surprisingly, vancomycin showed a high MIC (non-susceptible, 16 µg/mL). Additionally, due to disease rarity, there are no defined treatment guidelines for any *Paenibacillus* species. Previous research on antimicrobial susceptibility and resistance for isolates of this genus has utilized the interpretative criteria for *Bacillus* strains to determine breakpoints (2, 31, 32).

### Conclusion

This case documents the third reported human *Paenibacillus pueri* infection and the first reported *P. pueri* aortic pseudoaneurysm infection. Furthermore, it demonstrates the difficulties in identifying *P. pueri*. As environmental organisms, *Bacillus* spp. and related genera such as *Paenibacillus* spp*.* are often contaminants; however, rare cases of pathogenicity are seen, and repeated isolation in separate specimens should raise suspicion for further clinical evaluation and management.
